# Optimizing Quality of Care for Elderly Tuberculosis Patients in Shanghai, China: Insights from Patient Cascade of Care and Patient Pathway Analysis

**DOI:** 10.3390/tropicalmed11020052

**Published:** 2026-02-12

**Authors:** Yutong Han, Lixin Rao, Yu Huang, Qi Zhao, Xin Shen, Biao Xu

**Affiliations:** 1 Department of Epidemiology, School of Public Health, Fudan University, Shanghai 200032, China; ythan22@m.fudan.edu.cn (Y.H.); 22211020139@m.fudan.edu.cn (Y.H.); zhaoqi@shmu.edu.cn (Q.Z.); 2 Key Laboratory of Health Technology Assessment, National Health Commission of the People’s Republic of China, Fudan University, Shanghai 200032, China; 3 Division of Tuberculosis and HIV/AIDS Prevention, Shanghai Municipal Center for Disease Control and Prevention, Shanghai 201107, China; raolixin@scdc.sh.cn (L.R.); shenxin@scdc.sh.cn (X.S.); 4 Shanghai Institutes of Preventive Medicine, Shanghai 201107, China

**Keywords:** tuberculosis, quality of care, patient care cascade analysis, patient pathway analysis

## Abstract

With population aging, the burden of tuberculosis (TB) among the elderly is rising. Older adults are at high risk of TB but susceptible to poor TB care. In this study, we enrolled TB patients aged over 60 years registered in Shanghai during 2019–2021. A seven-step care cascade from estimated TB burden in the community to treatment outcomes was constructed to quantify retention and attrition at each step of TB health service. Patient pathway analysis was carried out in two districts of Shanghai to describe patients’ care-seeking behaviors, service coverage, and diagnosis delays. Across the care cascade, the largest gaps occurred from symptom onset to care seeking (11.3%) and from treatment initiation to completion (10.7%). Male sex, older age, and mycobacterium tuberculosis positivity were associated with treatment discontinuation and unfavorable outcomes. The patient pathway analysis revealed that first contact at lower-level or non-TB-designated hospitals was associated with more complex pathways and may contribute to diagnostic delays. These findings highlight the need to promote proactive care seeking upon symptoms, strengthen targeted adherence support for older people, and improve diagnostic capacity and referral efficiency at lower-level health facilities.

## 1. Introduction

Tuberculosis (TB) remains a major global public health concern. In 2023, an estimated 8.2 million people developed TB worldwide [1]. After three years of being surpassed by COVID-19, TB has reclaimed its position as the leading infectious disease killer. Despite notable progress in reducing the global TB burden over past decades, the world did not attain the first target of the WHO End TB Strategy in 2020. The pace of decline has varied across age groups, with the slowest progress observed among older adults [2]. The disease burden has shifted from younger to older age groups [3]. Given the rapidly aging global population and limited age-specific guidance, TB prevention and control strategies for older adults warrant greater attention [4]. China has the world’s largest older population [5]. By 2050, the number of people aged 65 years and above is projected to increase further to 395 million [6]. This demographic shift, together with increasing life expectancy and risk of TB reactivation, poses new challenges for TB prevention and control among older adults in China.

The diagnosis and management of TB in older adults are more complex compared with younger populations [7,8]. Older adults are both susceptible to new TB infections and are at increased risk of reactivation from long-term infection. Physical frailty and limited mobility may impede timely care seeking. Elderly TB patients often present with atypical clinical symptoms which could lead to missed or delayed diagnosis. To improve the quality of care for older adults, the WHO has advocated for the development of age-friendly health systems [9] and has identified “patient-centered care for all people with TB” as a key pillar of its post-2015 End TB Strategy [10]. Improving care quality and treatment outcomes requires clear identification of gaps along the TB care continuum and explicit prioritization of interventions [11].

To measure the quality of TB care, this study applied two complementary quantitative approaches to get a comprehensive understanding of the care continuum from population- and individual-level perspectives [12]. The first is patient care cascade analysis (PCA), which could depict the proportion of people who reach important milestones in the care continuum, from being unnoticed in the population to achieving optimal treatment outcomes [13]. The gaps between steps quantify attrition of the patient population and identify risk factors for patients dropping out of care on their way to achieving a cure [14]. The second is patient pathway analysis (PPA), which offers an individual-level perspective on the care-seeking experiences of patients. PPA begins where TB patients initiate seeking healthcare and follows them by recording the times and places of healthcare visits for each patient until they receive a TB diagnosis and/or achieve treatment completion [12]. PPA utilizes individual patient data to determine the alignment of patient care seeking and service availability, which could reveal where and for how long TB diagnosis and treatment delay occur during care seeking [15]. Although PCA and PPA have been increasingly applied in studies of TB, evidence focused on elderly TB patients remains limited [16,17].

This study aimed to evaluate the quality of TB care among older adults in Shanghai, China, by applying stepwise PCA spanning from estimated TB burden in the community to the treatment outcomes of patients using data from the National Tuberculosis Information Management System (TBIMS) and PPA focused on pre-diagnosis care-seeking pathways in diagnosed TB patients through linking TBIMS data and Shanghai Health Information Network (SHIN) data. The findings on gaps in TB care cascade and diagnosis delay are expected to inform age-specific TB prevention and care strategies.

## 2. Materials and Methods

### 2.1. Study Setting

Shanghai is an economically developed, densely populated, and rapidly aging city in eastern China, comprising 16 administrative districts covering both urban and suburban areas. In 2024, people aged over 60 years accounted for 37.37% of the household-registered population. TB incidence in Shanghai has declined steadily in recent years, ranking among the lowest in the country. However, the proportion of older adults among TB patients increased in the past decade. In 2023, the overall TB notification rate was 19.55 per 100,000, with the highest in those aged ≥65 years (48.47 per 100,000) [18].

According to the national TB prevention and control program, all TB patients must be registered in TBIMS, which collects the clinical and demographic information of patients. In parallel, Shanghai operates a mature and reliable electronic health information platform, the SHIN, which records information on all outpatient visits and hospitalizations including date, place, diagnosis, medication, operation, etc., together with patient demographic data from all health facilities in Shanghai. The two platforms are linked by the municipal health data center to generate pseudonymized, de-identified datasets for analysis, which provides a valuable opportunity to understand real-world care-seeking behaviors among TB patients.

### 2.2. Patients Care Cascade Analysis

TB patients aged over 60 years registered in TBIMS in Shanghai from 1 January 2019 to 31 December 2021 were enrolled in the PCA. Patients were excluded if they had extrapulmonary TB and non-tuberculosis mycobacterial infections. Information on age, sex, registered residence, TB bacteriological diagnosis (positive, negative, or not documented), comorbidities (diabetes and HIV infection), treatment history of TB (new or previously treated), date of symptom onset, date of diagnosis, and treatment outcomes (cured, treatment completed, treatment failed, lost to follow-up, or died) was collected from TBIMS. For this study, treatment success was defined as either being cured or treatment completed, while all other outcomes were classified as unfavorable.

A seven-step cascade of TB care was constructed, comprising the following steps: (1) patients in the community; (2) patients who seek healthcare; (3) patients who access TB testing; (4) patients who receive a TB diagnosis; (5) patients who initiate treatment; (6) patients who complete treatment; and (7) patients who achieve treatment success [17]. In this study, the number of elderly patients was estimated from steps 1 to 3. The number of patients in the community (step 1) was estimated from the number of diagnosed elderly TB patients recorded in the TBIMS (corresponding to Step 4) divided by the case detection rate (CDR) for China reported in the WHO Global TB Report [19,20,21]. Because WHO reports CDR at the national level only, we used China’s annual CDRs as a proxy for Shanghai and assumed that the national detection rate was applicable to older adults in Shanghai. In specific, the CDRs were 0.88 (95% uncertainty interval (UI): 0.73–1.10) in 2019, 0.75 (95% UI: 0.62, 0.92) in 2020, and 0.75 (95% UI: 0.62–0.92) in 2021. The number of patients who sought healthcare (step 2) was calculated as the number of TB patients receiving TB testing divided by the proportion of patients seeking care for TB symptoms. In this study, a 94.8% care-seeking proportion was applied based on a parallel survey conducted among 350 elderly TB patients in Shanghai. The number of patients who access TB testing (step 3) was calculated as the number of diagnosed TB patients divided by diagnostic sensitivity. In this care cascade, “accessed TB testing” (step 3) refers to receipt of diagnostic examination for active TB, including bacteriological tests (e.g., sputum smear microscopy, culture, and molecular testing) and chest imaging. Given that GeneXpert MTB/RIF was routinely implemented in Shanghai during 2019–2021, we assumed that all notified TB patients had received GeneXpert MTB/RIF testing as part of the diagnostic examination. Therefore, we used the diagnostic sensitivity of GeneXpert MTB/RIF as the key parameter for this step. Based on the findings of Feng et al., the sensitivity of GeneXpert MTB/RIF in detecting active TB was 0.94 (95% confidence interval (CI): 0.92–0.95) among Chinese people [22]. Steps 4 to 7 were obtained directly from TBIMS.

Since steps 1 to 3 were indirectly estimated, we quantified uncertainty using probabilistic sensitivity analysis using Monte Carlo simulation with 10,000 iterations. In each iteration, the annual CDR, diagnostic sensitivity, and the care-seeking proportion were sampled from Beta distributions. These distributions were parameterized based on the point estimates and their respective uncertainty ranges, as described above. Steps 1–3 were recalculated in each iteration, and the 95% UIs were summarized by the 2.5th and 97.5th percentiles of the simulated distributions.

### 2.3. Patient Pathway Analysis

Of the 16 districts in Shanghai, the 10 central districts are fully urbanized for residence and business, while the remaining 6 suburban districts are used primarily for agriculture and industry. The PPA study was carried out in two districts of Shanghai, one being an urban area (District A) and the other a suburban area (District B). These districts were selected to provide a comprehensive assessment of the TB healthcare system’s performance in Shanghai, considering the differences in economic development, healthcare infrastructure, and patient care-seeking behavior between these areas. The study subjects were all eligible adults aged ≥60 years with TB registered in TBIMS during 2019–2021 in these districts. Individual care-seeking data corresponding to these patients were obtained from the SHIN. The data were retrieved for the period from the onset of TB-related symptoms to diagnosis, including the health facility for first visit, the health facility for TB diagnosis, all other health facilities visited, and the dates of visits along the care-seeking pathway. There was no need for patient self-reporting. The first visit for TB symptoms was identified in SHIN by symptom records (e.g., cough ≥ 2 weeks, sputum production, hemoptysis, fever, night sweats, weight loss, and/or chest pain).

According to the previous literature [23,24], delays in TB diagnosis were presented as patient delay, health system delay, and diagnosis delay. Patient delay was defined as a time interval longer than 14 days from the onset of symptoms to the first visit to a health facility. Health system delay was a time interval longer than 14 days from the first health facility visits for TB symptoms to the diagnosis of TB. Diagnosis delay was the sum of patient delay and health system delay.

China has a three-tier health system from primary health centers to secondary and tertiary hospitals. Under this system, China’s TB medical care is handled by TB-designated hospitals in each county/district (mostly at the secondary level) and above the administration division. In Shanghai, each district has a TB-designated hospital, together with several tertiary hospitals that provide TB health services. Suspected TB cases identified in non-TB-designated hospitals should be referred accordingly.

### 2.4. Data Analysis

Descriptive data were summarized and presented as frequencies and percentages. The number of patients along the TB care cascade were displayed as an onion plot to visualize attrition between each step. The analysis of health facilities visited by the patients was presented using a stacked column chart. The first four patient visits were selected for visualization of patient flow, and Sankey diagrams were used to illustrate the flow of patients through different healthcare pathways [25]. Multivariable logistic regression was employed to identify factors associated with gaps in the cascade and delays in TB diagnosis. According to the previous relevant literature and variables collected in the TBIMS and SHIN [17,25], the following variables were taken as covariates: district of registration (suburban area vs. urban area), sex (male vs. female), age group (60–69, 70–79, vs. >80 years), registered residence (Shanghai vs. other regions), bacteriological diagnosis status (positive, negative vs. not documented), treatment history of TB (new vs. previously treated), history of diabetes (yes vs. no), first-visit health facility level (primary, secondary vs. tertiary), and first-visit health facility type (TB-designated vs. non-TB-designated). All covariates were entered into multivariable models simultaneously. Multicollinearity was assessed using variance inflation factors (VIFs). Crude odds ratios (cORs) from univariable models and adjusted odds ratios (aORs) from multivariable models are reported. For the care cascade analysis based on TBIMS, there were no missing values for the covariates used. For the PPA, two older patients reported in TBIMS could not be linked to corresponding care-seeking records in SHIN and thus were excluded. All statistical analyses were performed using R 4.4.2 software, and the significance level of a statistical test was 0.05.

## 3. Results

### 3.1. Cascade of TB Care Among the Elderly in Shanghai, 2019–2021

**Figure 1:**
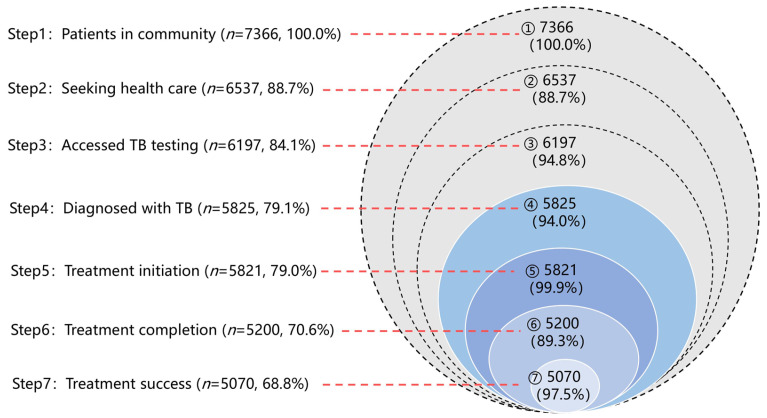
Care cascade for elderly patients diagnosed with tuberculosis in Shanghai, 2019–2021. The left panel shows the number of individuals and cumulative percentage at each step, using the estimated incident TB cases (*n* = 7366) as the denominator. Numbers inside the circles indicate the count at each step, and the percentages indicate stepwise retention relative to the previous step.

From 2019 to 2021, 5825 older adults with TB were recorded in Shanghai; 4331 (74.4%) were male, 88.4% were local residents, and 88.5% were newly diagnosed (Table S1). The care cascade is shown in Figure 1. Based on the annually reported CDRs, an estimated 7366 (95% UI: 6573–8203) incident TB cases occurred in the community. Of these, 88.7% (*n* = 6537, 95% UI: 6384–6771) sought healthcare, and 84.1% (*n* = 6197, 95% UI: 6121–6322) received TB diagnostic services. Among those diagnosed, 99.9% initiated treatment, and 89.3% completed the treatment. Overall, 87.0% (5070/5825) of diagnosed patients achieved treatment success. Substantial patient attrition was observed at two gaps: 11.3% of patients failed to seek care (step 1 to step 2), and 10.7% of patients who initiated treatment did not complete it (step 5 to step 6).

### 3.2. Factors Associated with the Attrition of Elderly Patients in the TB Care Cascade

Among 5825 elderly TB patients, 625 (10.7%) did not complete treatment, and 755 (11.9%) had unfavorable outcomes. Unfavorable outcomes comprised non-TB death (415/755, 55.0%), TB-related death (112/755, 14.8%), loss to follow-up (98/755, 13.0%), and treatment failure (130/755, 17.2%). Multivariable logistic regression showed that sex, age, and bacteriological diagnosis were associated with both treatment completion and outcomes (all *p* < 0.05; Table 1). There was no multicollinearity between covariates in the multivariate model (all VIFs < 2). Female patients and those with negative bacteriological diagnosis of mycobacterium tuberculosis were less likely to discontinue treatment and experience unfavorable outcomes (aOR < 1). Patients aged ≥70 years, particularly those ≥80 years, were more likely to discontinue treatment and to have unfavorable outcomes (aOR > 1) (Table 1). Moreover, previously treated patients were more likely to have unfavorable outcomes. The cORs from the univariable logistic regression for failure to complete treatment and unfavorable treatment outcome are presented in Table S2.

### 3.3. Service Flow Among Different Health Facilities for Elderly TB Patients

**Figure 2:**
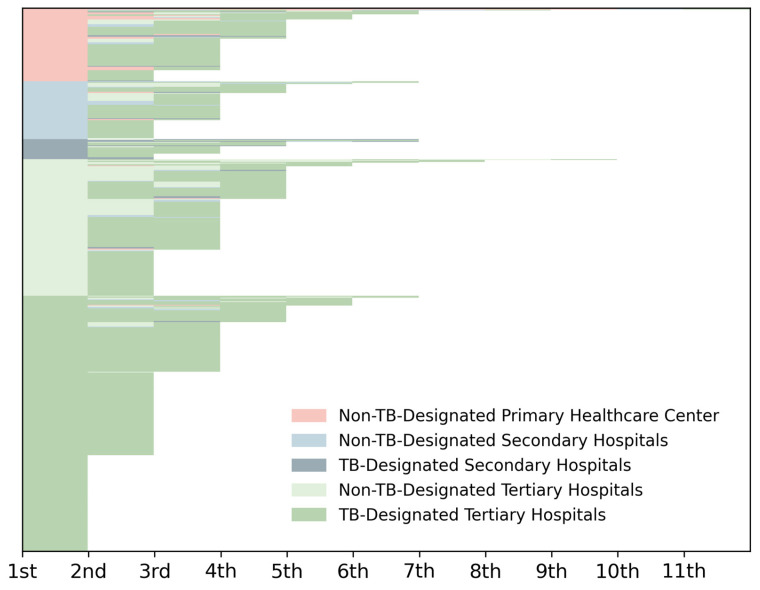
Health facilities visited by elderly patients with TB before diagnosis. Different gradations of color represent different levels and categories of health facilities. Each medical visit among the 600 participants is stratified by the categories and levels of health facilities. The bottom text refers to the times of medical visits. Each row presents one case.

All eligible elderly TB patients recorded in TBIMS during 2019–2021 from two representative districts were enrolled in the PPA (*n* = 600; Table S3). Figure 2 illustrates the distribution of health facility visits before diagnosis, ranging from 1 to 11 visits (median 3.0, IQR 2.0–3.0). Specifically, 108 patients (108/600, 18.0%) were diagnosed at their first visit, 181 (181/600, 30.2%) at the second, 184 (184/600 30.7%) at the third, 90 (90/600, 15.0%) at the fourth, and 37 (37/600, 6.2%) after ≥5 visits. In terms of initial health facilities visited by patients, 13.3% (80/600) of visits were at primary non-TB-designated healthcare centers, 10.7% (64/600) at secondary general hospitals, and 3.7% (22/600) at secondary district TB-designated hospitals. About 72.3% (434/600) of patients visited tertiary hospitals directly, 34.8% (151/434) visited non-designated general hospitals, and 65.2% (283/434) visited TB-designated hospitals.

**Figure 3:**
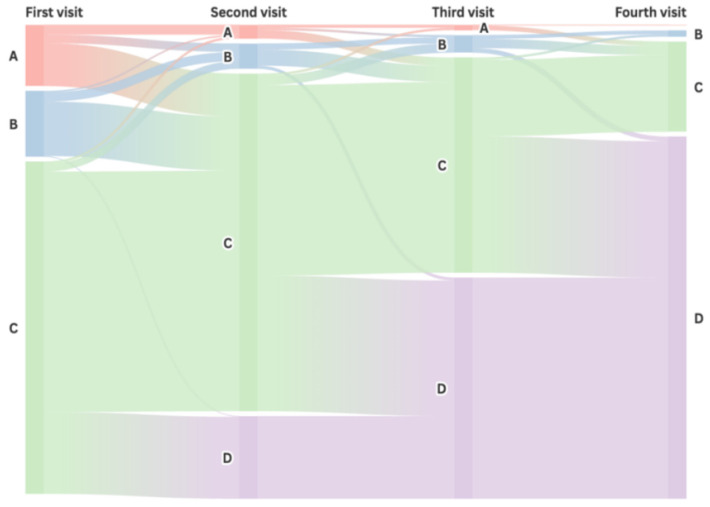
Sankey diagram of the patients’ visits. A—red part refers to patients who visited primary healthcare centers; B—blue part refers to patients who visited secondary health facilities (including TB-designated and non-TB-designated hospitals); C—green part refers to patients who visited tertiary health facilities (including TB-designated and non-TB-designated hospitals); D—purple part refers to patients who were diagnosed with TB at health facilities.

The Sankey diagram of patient visits (Figure 3) illustrates that 563 patients (93.8%) were diagnosed with TB within four visits while the remaining 37 patients (6.2%) were still navigating different levels of health facilities without a TB diagnosis. Of the patients whose first visit was at tertiary hospitals, 361 (83.2%) were diagnosed within 1–3 visits. For those who initially sought care at secondary hospitals, 64 (74.4%) were diagnosed within 1–3 visits. Of the patients who first visited primary healthcare centers, 67 (83.8%) were referred to higher-level health facilities by their second visit, and 43 (53.8%) were diagnosed within 2–3 visits. The remaining patients had two or more visits at primary healthcare facilities before being referred to higher-level hospitals where they were finally diagnosed.

### 3.4. Factors Associated with the Delays in TB Care Among Elderly TB Patients

Among 600 older adults with TB, patient delay and health system delay occurred in 11.8% and 49.7%, respectively, summed to a 27.0% diagnosis delay. No multicollinearity was observed in the multivariable logistic model (all VIFs < 2). Multivariable analysis indicated that patients with negative bacteriological diagnosis were significantly less likely to experience patient delay (aOR = 0.476, 95% CI: 0.252–0.897) (Table 2). Health system delay was more likely among patients aged ≥80 years and those with bacteriologically negative TB (aOR > 1), whereas visiting a TB-designated hospitals first was associated with a lower likelihood of health system delay (aOR = 0.292, 95% CI: 0.197–0.431). Regarding diagnosis delay, patients who were non-Shanghai residents were more likely to experience delays (aOR = 2.349, 95% CI: 1.390–3.969). In contrast, patients who initially visited secondary (aOR = 0.497, 95% CI: 0.251–0.987) or tertiary hospitals (aOR = 0.502, 95% CI: 0.277–0.909) were less likely to experience a diagnosis delay than those initiating care at primary healthcare centers. The cORs from univariable logistic regression are presented in Table S4.

## 4. Discussion

This study provides a comprehensive assessment of TB quality of care for elderly patients in Shanghai by combining a population-level PCA with an individual-level PPA based on real-world data. From the perspectives of TB management in population and individual care experiences, we delineated gaps across the care continuum and characterized the complexity of navigation through the three-tier health system. The findings highlight key bottlenecks in quality of care and inform priorities for targeted interventions, including improved case finding, tailored treatment adherence support, and more efficient referral pathways.

The analysis of TB care cascade among elderly patients revealed two major gaps of patient attrition. At the pre-diagnosis stage, more than one in ten people who had TB disease did not seek care. At the post-diagnosis stage, among those who initiated anti-TB treatment, only 89.3% completed therapy. These gaps point towards the key priorities for TB programs [26]. In China, the current strategy for TB detection is passive case finding (PCF), in which symptomatic patients actively seek care at health facilities. A reliance on PCF could result in infectious TB patients being overlooked, which could lead to ongoing transmission of infection within families and communities [27]. Low case detection happens under PCF when access to healthcare is problematic due to economic disadvantages, geographic difficulty, negligence of symptoms, and reduced mobility, especially in the elderly population [28].

Active case finding (ACF) has been increasingly recognized as an important complementary strategy to PCF. A study conducted in eastern China demonstrated that repeated sequential ACF screening might decrease the TB epidemic among older adults [29]. A concern with ACF is the cost-effectiveness in the low and middle TB burden population [30]. In Shanghai, TB incidence in the elderly population is about 48 per 100,000 [18]. Older adults are more likely to dismiss TB symptoms because of chronic bronchitis and smoking-induced symptoms. Moreover, poor TB awareness and stigma may also lead to delayed care seeking and TB diagnosis [7,31]. Thus, the optimal strategies may involve enhancing people’s knowledge of TB symptoms to speed care seeking, informing risks of TB in people with chronic disease, actively finding TB cases through routine health checkups, and improving economic and geographic access to healthcare for the elderly population [31,32,33].

Treatment completion is vital to favorable outcomes. This study found that sex, age, and bacteriological diagnosis were associated with both treatment completion and treatment outcomes among elderly TB patients. Cognitive decline and frailty, together with chronic disease comorbidity, could increase older patients’ susceptibility to adverse drug reactions and reduce their treatment tolerance, thereby undermining adherence to TB treatment. Women generally show better health awareness and more proactive care seeking [34], whereas alcohol and tobacco use among men may increase the risk of treatment interruption [35]. Patients with negative bacteriological diagnosis tend to have milder symptoms and fewer adverse events, which may facilitate treatment adherence [7]. From the health system perspective, older adults and men may be more susceptible to the poor accessibility of health services and adherence supports [7,36]. Accordingly, primary healthcare centers should provide tailored health education and adherence support for male and older adults to promote timely symptom-based care seeking and treatment adherence [37]. In addition, evidence from other settings suggests that TB team-based care, encompassing structured care with trained staff, dedicated outpatient clinics, and proactive follow-up, could improve adherence and treatment outcomes, especially among vulnerable populations [38]. Thus, reinforcement of team-based TB services could be a pragmatic option to complement China’s designated-hospital strategy, particularly for older adults who need more intensive follow-up and support.

In this study, the PPA results further revealed the complexity of diagnostic trajectories among elderly TB patients. A greater number of visits reflects a longer and more complex diagnostic pathway, which may indicate prolonged diagnosis and higher healthcare costs, while also reflect risks for community transmission [39]. Challenges with navigation between health facilities is one of the major barriers associated with not seeking care and patient loss along the TB care pathway [40]. Under the three-tier health system, non-TB-designated hospitals in China should refer suspected TB patients to designated hospitals [25]. The centralization of TB diagnosis and treatment plays a crucial role in patient management [41,42]. However, this system may weaken diagnostic capacity among clinicians in non-TB-designated health facilities, especially primary health centers, where many elderly patients prefer to seek care. Building the capacity for TB care and patient management at primary and non-TB-designated facilities would contribute to timely diagnosis and efficient referrals within existing national TB control strategies [24]. Optimization of TB referral mechanisms for older patients and continuous medical education on TB for clinicians are urgently required.

This study has certain limitations. First, without a population survey, the pre-diagnosis steps in the care cascade had to be estimated using parameters from previous studies and national surveillance, which may introduce underestimation or overestimation bias. Given Shanghai’s relatively low TB burden and comparatively advanced healthcare system, applying the national CDR may overestimate the community TB burden. In addition, because older adults often present with atypical symptoms and have a higher burden of comorbidities, case detection among older adults may be lower than that in the general population. Second, some patients might have sought care outside Shanghai, which could not be captured by SHIN, especially for non-Shanghai residents. Third, other potential influencing factors, such as comorbidities with chronic obstructive pulmonary disease and other diseases, socioeconomic status, education level, disease severity, and geographic accessibility to hospitals, were not collected in this study. Future research should incorporate these variables to provide a more complete assessment of determinants of TB care among elderly patients. Finally, since this study was conducted in Shanghai, a high-resource megacity with a dense healthcare network and mature health infrastructure, the generalizability of our results to lower-resource or rural settings may be constrained. The step-specific cascade levels depend on local TB control capacity and service availability, so the results may be more generalizable for relatively developed cities and countries where TB burden in an aging population is a public health concern. Because the PPA was undertaken within China’s three-tier and TB-designated hospital system, the pathway patterns may be less applicable to settings with different health system architectures. Nonetheless, the overarching findings that aging populations require integrated, patient-centered, and age-friendly TB care are globally relevant.

## 5. Conclusions

In conclusion, the greatest attrition among older adults occurred at two milestones across the TB care cascade: care seeking and treatment completion. TB program should prioritize age-specific health promotion to improve recognition of TB symptoms and promote timely and proactive care seeking. Targeted treatment adherence support, particularly for male, older-age, and bacteriologically positive patients may help improve treatment outcomes. The complexity of care-seeking journeys underscores the importance of a referral mechanism in the TB care system. Improving diagnostic capacity at lower-level health facilities and establishing efficient referrals are critical to timely TB diagnosis. Taken together, these actions may help reduce TB burden in aging populations and support progress towards the WHO End TB targets.

## Figures and Tables

**Table 1:** Multivariate analysis for patient attrition gaps in elderly patients with TB.

Factors	Failed to Complete Treatment (*n* = 625)		Unfavorable Treatment Outcome (*n* = 755)	
	*n* (%)	aOR (95% CI) *	*n* (%)	aOR (95% CI) *
Registration district				
Suburban area	424 (7.2)	Ref.	504 (12.8)	Ref.
Urban area	201 (10.6)	0.944 (0.783, 1.236)	251 (13.2)	1.000 (0.843, 1.183)
Sex				
Male	523 (12.1)	Ref.	635 (14.7)	Ref.
Female	102 (6.8)	0.535 (0.424, 0.670)	120 (8.0)	0.530 (0.427, 0.652)
Age, years				
60–69	170 (5.8)	Ref.	258 (8.8)	Ref.
70–79	192 (10.3)	1.824 (1.469, 2.266)	230 (12.4)	1.428 (1.179, 1.727)
≥80	263 (25.2)	5.355 (4.334, 6.632)	267 (25.6)	3.479 (2.865, 4.226)
Registered residence				
Shanghai	578 (11.2)	Ref.	690 (13.4)	Ref.
Other regions	47 (6.9)	0.761 (0.547, 1.035)	65 (9.6)	0.846 (0.637, 1.107)
Bacteriological diagnosis				
Positive	447 (13.2)	Ref.	571 (16.8)	Ref.
Negative	125 (6.7)	0.545 (0.439, 0.673)	131 (7.1)	0.420 (0.342, 0.513)
Not documented	53 (9.2)	0.642 (0.466, 0.867)	53 (9.2)	0.507 (0.371, 0.681)
Treatment history of TB				
New	550 (10.7)	Ref.	632 (12.3)	Ref.
Previously treated	75 (11.2)	0.953 (0.726, 1.236)	123 (18.4)	1.465 (1.171, 1.821)
History of diabetes				
No	507 (10.4)	Ref.	615 (12.6)	Ref.
Yes	118 (2.4)	1.176 (0.938, 1.465)	140 (14.7)	1.104 (0.897, 1.353)

* Adjusted by registration district, age, sex, registered residence, bacteriological diagnosis, treatment history of TB, and history of diabetes. aOR = adjusted odds ratio; CI = confidence interval.

**Table 2:** Multivariate analysis of delays in TB care among elderly patients with TB.

Factors	Patient Delay (*n* = 71)		Health System Delay (*n* = 298)		Diagnosis Delay (*n* = 162)	
	*n* (%)	aOR (95% CI) *	*n* (%)	aOR (95% CI) ^#^	*n* (%)	aOR (95% CI) ^#^
Registration district						
Urban area	27 (9.0)	Ref.	138 (46.2)	Ref.	64 (21.4)	Ref.
Suburban area	44 (14.6)	1.552 (0.906, 2.660)	160 (53.2)	1.222 (0.849, 1.760)	98 (32.6)	1.375 (0.925, 2.045)
Sex						
Male	52 (11.3)	Ref.	222 (48.2)	Ref.	120 (26.0)	Ref.
Female	19 (13.7)	1.327 (0.742, 2.375)	76 (54.7)	1.270 (0.841, 1.919)	42 (30.2)	1.168 (0.756, 1.805)
Age, years						
60~	39 (13.0)	Ref.	133 (44.3)	Ref.	75 (25.0)	Ref.
70~	21 (10.4)	0.726 (0.408, 1.292)	107 (53.0)	1.289 (0.877, 1.896)	60 (29.7)	1.164 (0.767, 1.765)
≥80	11 (11.2)	0.881 (0.424, 1.830)	58 (59.2)	1.835 (1.111, 3.031)	27 (27.6)	1.211 (0.707, 2.074)
Registered residence						
Shanghai	55 (10.6)	Ref.	254 (48.9)	Ref.	128 (24.7)	Ref.
Other regions	16 (19.8)	1.858 (0.966, 3.573)	44 (54.3)	1.300 (0.769, 2.196)	34 (42.0)	2.349 (1.390, 3.969)
Bacteriological diagnosis						
Positive	53 (14.4)	Ref.	172 (46.9)	Ref.	102 (27.8)	Ref.
Negative	14 (7.3)	0.476 (0.252, 0.897)	99 (51.6)	1.544 (1.046, 2.280)	45 (23.4)	0.876 (0.571, 1.343)
Not documented	4 (9.8)	0.645 (0.217, 1.914)	27 (65.9)	2.264 (1.100, 4.660)	15 (36.6)	1.457 (0.724, 2.931)
Treatment history of TB						
New	64 (11.8)	Ref.	271 (50.0)	Ref.	150 (27.7)	Ref.
Previously treated	7 (12.1)	0.951 (0.405, 2.234)	27 (46.6)	1.085 (0.603, 1.953)	12 (20.7)	0.711 (0.359, 1.407)
History of diabetes						
No	53 (10.7)	Ref.	247 (49.8)	Ref.	132 (26.6)	Ref.
Yes	18 (17.3)	1.808 (0.990, 3.303)	51 (49.0)	1.075 (0.681, 1.699)	30 (28.8)	1.251 (0.766, 2.043)
First-visit health facility level						
Primary	-	-	55 (68.8)	Ref.	34 (42.5)	Ref.
Secondary	-	-	52 (59.1)	0.976 (0.495, 1.922)	22 (25.0)	0.497 (0.251, 0.987)
Tertiary	-	-	191 (44.2)	0.803 (0.443, 1.456)	106 (24.5)	0.502 (0.277, 0.909)
First-visit health facility type						
Non-TB-designated	-	-	195 (65.7)	Ref.	94 (31.6)	Ref.
TB-designated	-	-	103 (34.0)	0.292 (0.197, 0.431)	68 (22.4)	0.812 (0.526, 1.253)

* Adjusted by registration district, age, sex, registered residence, bacteriological diagnosis, treatment history of TB, history of diabetes. ^#^ Adjusted by registration district, age, sex, registered residence, bacteriological diagnosis, treatment history of TB, history of diabetes, first-visit health facility level, and first-visit health facility type. aOR = adjusted odds ratio; CI = confidence interval.

## Data Availability

The datasets used and analyzed during the current study are available from the corresponding author upon reasonable request.

## Supplementary Materials

The following supporting information can be downloaded at: https://www.mdpi.com/article/10.3390/tropicalmed11020052/s1, Table S1: Demographic and clinical characteristics of elderly TB patients in Shanghai, 2019–2021; Table S2: Univariable analysis of patient attrition gaps in elderly patients with TB. Table S3: Demographic and clinical characteristics of elderly TB patients from two districts in Shanghai, 2019–2021. Table S4: Univariable analysis of delays in TB care among elderly patients with TB.

## Abbreviations

The following abbreviations are used in this manuscript:

TB	Tuberculosis
TBIMS	National Tuberculosis Information Management System
PCA	Patient care cascade analysis
PPA	Patient pathway analysis
SHIN	Shanghai Health Information Network
CDR	Case detection rate
VIFs	Variance inflation factors
cORs	Crude odds ratios
aORs	Adjusted odds ratios
CI	Confidence interval
UI	Uncertainty interval
ACF	Active case finding
PCF	Passive case finding
