# Supplementary material for: Optimizing Quality of Care for Elderly Tuberculosis Patients in Shanghai, China: Insights from Patient Cascade of Care and Patient Pathway Analysis
Source: Trop Med Infect Dis. 2026 Feb 12;11(2):52. doi: 10.3390/tropicalmed11020052 (PMC12945059; doi:10.3390/tropicalmed11020052)
Supplement: Supplementary file 1 [file tropicalmed-11-00052-s001.zip › tropicalmed-4058977-supplementary.docx]

| Table S1. Demographic and clinical characteristics of elderly TB patients in Shanghai, 2019–2021 | | | |
| --- | --- | --- | --- |
| Characteristics | | *n* | % |
| Time of registration | 2019 | 2012 | 34.5% |
|  | 2020 | 1827 | 31.4% |
|  | 2021 | 1986 | 34.1% |
| Registration district | Urban areas | 1900 | 32.6% |
|  | Suburban areas | 3925 | 67.4% |
| Sex | Male | 4331 | 74.4% |
|  | Female | 1494 | 25.6% |
| Age group, years | 60–69 | 2926 | 50.2% |
|  | 70–79 | 1857 | 31.9% |
|  | ≥80 | 1042 | 17.9% |
| Registered residence | Shanghai | 5147 | 88.4% |
|  | Other regions | 678 | 11.6% |
| Bacteriological diagnosis | Positive | 3395 | 58.3% |
|  | Negative | 1855 | 31.8% |
|  | Not documented | 575 | 9.9% |
| Treatment history of TB | New | 5156 | 88.5% |
|  | Previously treated | 669 | 11.5% |
| History of diabetes | Yes | 955 | 16.4% |
|  | No | 4870 | 83.6% |
| HIV | Yes | 19 | 0.3% |
|  | No | 5806 | 99.7% |

**Supplementary Materials**

| Table S2. Univariable analysis of patient attrition gaps in elderly patients with TB | | | | |
| --- | --- | --- | --- | --- |
| Factors | Failed to complete treatment | | Unfavorable treatment outcome | |
|  | (*n*=625) | | (*n=*755) | |
|  | *n* (%) | cOR (95% CI) | *n* (%) | cOR (95% CI) |
| Registration district |  |  |  |  |
| Suburban area | 424 (7.2) | Ref. | 504 (12.8) | Ref. |
| Urban area | 201 (10.6) | 0.977 (0.817, 1.165) | 251 (13.2) | 0.990 (0.834, 1.171) |
| Sex |  |  |  |  |
| Male | 523 (12.1) | Ref. | 635 (14.7) | Ref. |
| Female | 102 (6.8) | 0.534 (0.426, 0.662) | 120 (8.0) | 0.507 (0.408, 0.625) |
| Age, years |  |  |  |  |
| 60–69 | 170 (5.8) | Ref. | 258 (8.8) | Ref. |
| 70–79 | 192 (10.3) | 1.869 (1.508, 2.320) | 230 (12.4) | 1.641 (1.344, 2.005) |
| ≥80 | 263 (25.2) | 5.473 (4.448, 6.752) | 267 (25.6) | 4.366 (3.587, 5.321) |
| Registered residence |  |  |  |  |
| Shanghai | 578 (11.2) | Ref. | 690 (13.4) | Ref. |
| Other regions | 47 (6.9) | 0.589 (0.427, 0.793) | 65 (9.6) | 0.653 (0.487, 0.860) |
| Bacteriological diagnosis |  |  |  |  |
| Positive | 447 (13.2) | Ref. | 571 (16.8) | Ref. |
| Negative | 125 (6.7) | 0.477 (0.386, 0.584) | 131 (7.1) | 0.415 (0.337, 0.507) |
| Not documented | 53 (9.2) | 0.670 (0.491, 0.895) | 53 (9.2) | 0.573 (0.421, 0.765) |
| Treatment history of TB |  |  |  |  |
| New | 550 (10.7) | Ref. | 632 (12.3) | Ref. |
| Previously treated | 75 (11.2) | 1.057 (0.813, 1.357) | 123 (18.4) | 1.395 (1.106, 1.744) |
| History of diabetes |  |  |  |  |
| No | 507 (10.4) | Ref. | 615 (12.6) | Ref. |
| Yes | 118 (2.4) | 1.213 (0.976, 1.497) | 140 (14.7) | 1.210 (0.983, 1.481) |
| cOR = crude odds ratio; CI =confidence interval. | | | | |

| Table S3. Demographic and clinical characteristics of elderly TB patients from two districts in Shanghai, 2019–2021 | | | |
| --- | --- | --- | --- |
| Characteristics | | n | % |
| Time of registration | 2019 | 216 | 36.0 |
|  | 2020 | 199 | 33.2 |
|  | 2021 | 185 | 30.8 |
| Sex | Male | 461 | 76.8 |
|  | Female | 139 | 23.2 |
| Age group, years | 60–69 | 300 | 50.0 |
|  | 70–79 | 202 | 33.7 |
|  | ≥80 | 98 | 16.3 |
| Registered residence | Shanghai | 519 | 86.5 |
|  | Other regions | 81 | 13.5 |
| Bacteriological results | Positive | 367 | 61.2 |
|  | Negative | 192 | 32.0 |
|  | Not documented | 41 | 6.8 |
| Treatment history of TB | New | 542 | 90.3 |
|  | Previously treated | 58 | 9.7 |
| History of diabetes | Yes | 104 | 17.3 |
|  | No | 496 | 82.7 |
| HIV | Yes | 3 | 0.5 |
|  | No | 597 | 99.5 |
| Type of first visiting health facility | TB designated | 303 | 50.5 |
|  | Non-TB designated | 297 | 49.5 |
| Level of first visiting health facility | Primary | 80 | 13.3 |
|  | Secondary | 88 | 14.7 |
|  | Tertiary | 432 | 72.0 |
| Total number of health facility visits | **≤**3 visits | 474 | 79.0 |
|  | >3 visits | 126 | 21.0 |
| Patient delay^*^ | No | 529 | 88.2 |
|  | Yes | 71 | 11.8 |
| Health system delay^#^ | No | 302 | 50.3 |
|  | Yes | 298 | 49.7 |
| Diagnosis delay^&^ | No | 438 | 73.0 |
|  | Yes | 162 | 27.0 |
| Treatment outcome | Favorable | 516 | 86.0 |
|  | Unfavorable | 84 | 14.0 |
| * Patient delay was defined as a time interval longer than 14 days from the onset of symptoms to the first visit to a health facility.  # Health system delay was the interval longer than 14 days from the first visit to a health facility for TB symptoms to the diagnosis of TB.  & Diagnosis delay was the sum of patient delay and health system delay with a time interval exceeding 28 days. | | | |

| Table S4. Univariable analysis of delays in TB care among elderly patients with TB | | | | | | |
| --- | --- | --- | --- | --- | --- | --- |
| Factors | Patient delay | | Health system delay | | Diagnosis delay | |
|  | (*n*=71) | | (*n*=298) | | (*n*=162) | |
|  | *n* (%) | cOR (95%CI) | *n* (%) | cOR (95%CI) | *n* (%) | cOR (95%CI) |
| Registration district |  |  |  |  |  |  |
| Urban area | 27(9.0) | Ref. | 138(46.2) | Ref. | 64(21.4) | Ref. |
| Suburban area | 44(14.6) | 1.725 (1.044, 2.898) | 160(53.2) | 1.324 (0.961, 1.826) | 98(32.6) | 1.773 (1.231, 2.566) |
| Sex |  |  |  |  |  |  |
| Male | 52(11.3) | Ref. | 222(48.2) | Ref. | 120(26.0) | Ref. |
| Female | 19(13.7) | 1.245 (0.694, 2.154) | 76(54.7) | 1.299 (0.888, 1.904) | 42(30.2) | 1.230 (0.805, 1.859) |
| Age, years |  |  |  |  |  |  |
| 60~ | 39(13.0) | Ref. | 133(44.3) | Ref. | 75(25.0) | Ref. |
| 70~ | 21(10.4) | 0.776 (0.435, 1.351) | 107(53.0) | 1.414 (0.989, 2.025) | 60(29.7) | 1.268 (0.849, 1.889) |
| ≥80 | 11(11.2) | 0.846 (0.398, 1.672) | 58(59.2) | 1.821 (1.150, 2.907) | 27(27.6) | 1.141 (0.674, 1.893) |
| Registered residence |  |  |  |  |  |  |
| Shanghai | 55(10.6) | Ref. | 254(48.9) | Ref. | 128(24.7) | Ref. |
| Other regions | 16(19.8) | 2.077 (1.095, 3.768) | 44(54.3) | 1.241 (0.776, 1.992) | 34(42.0) | 2.210 (1.354, 3.578) |
| Bacteriological diagnosis |  |  |  |  |  |  |
| Positive | 53(14.4) | Ref. | 172(46.9) | Ref. | 102(27.8) | Ref. |
| Negative | 14(7.3) | 0.466 (0.243, 0.841) | 99(51.6) | 1.207 (0.851, 1.713) | 45(23.4) | 0.795 (0.527, 1.186) |
| Not documented | 4(9.8) | 0.640 (0.186, 1.680) | 27(65.9) | 2.186 (1.128, 4.415) | 15(36.6) | 1.499 (0.747, 2.912) |
| Treatment history of TB |  |  |  |  |  |  |
| New | 64(11.8) | Ref. | 271(50.0) | Ref. | 150(27.7) | Ref. |
| Previously treated | 7(12.1) | 1.025 (0.410, 2.220) | 27(46.6) | 0.871 (0.504, 1.498) | 12(20.7) | 0.682 (0.337, 1.282) |
| History of diabetes |  |  |  |  |  |  |
| No | 53(10.7) | Ref. | 247(49.8) | Ref. | 132(26.6) | Ref. |
| Yes | 18(17.3) | 1.749 (0.956, 3.083) | 51(49.0) | 0.970 (0.635, 1.481) | 30(28.8) | 1.118 (0.692, 1.771) |
| First-visit health facility level |  |  |  |  |  |  |
| Primary | - | - | 55(68.8) | Ref. | 34(42.5) | Ref. |
| Secondary | - | - | 52(59.1) | 0.657 (0.345, 1.235) | 22(25.0) | 0.451 (0.232, 0.863) |
| Tertiary | - | - | 191(44.2) | 0.360 (0.213, 0.593) | 106(24.5) | 0.440 (0.269, 0.725) |
| First-visit health facility type |  |  |  |  |  |  |
| Non-TB designated | - | - | 195(65.7) | Ref. | 94(31.6) | Ref. |
| TB-designated | - | - | 103(34.0) | 0.269 (0.192, 0.376) | 68(22.4) | 0.625 (0.433, 0.898) |
| cOR = crude odds ratio; CI =confidence interval. | | | | | | |
